# Vaccines as a Strategy to Control Trichinellosis

**DOI:** 10.3389/fmicb.2022.857786

**Published:** 2022-03-23

**Authors:** Bin Tang, Jian Li, Tingting Li, Yiting Xie, Wei Guan, Yanqing Zhao, Shuguo Yang, Mingyuan Liu, Daoxiu Xu

**Affiliations:** ^1^Department of Human Parasitology, School of Basic Medicine, Hubei University of Medicine, Shiyan, China; ^2^Key Laboratory of Zoonosis Research, Ministry of Education, Institute of Zoonosis, College of Veterinary Medicine, Jilin University, Changchun, China; ^3^Key Laboratory of Tropical Translational Medicine of Ministry of Education, Hainan Medical University, Haikou, China; ^4^Hainan Medical University-The University of Hong Kong Joint Laboratory of Tropical Infectious Diseases, Hainan Medical University, Haikou, China; ^5^Department of Pathogen Biology, Hainan Medical University, Haikou, China

**Keywords:** *Trichinella spiralis*, trichinellosis, antigens, vaccines, effectiveness

## Abstract

Trichinellosis caused by *Trichinella spiralis* is a worldwide food-borne parasitic zoonosis. Several approaches have been performed to control *T. spiralis* infection, including veterinary vaccines, which contribute to improving animal health and increasing public health by preventing the transmission of trichinellosis from animals to humans. In the past several decades, many vaccine studies have been performed in effort to control *T. spiralis* infection by reducing the muscle larvae and adult worms burden. Various candidate antigens, selected from excretory-secretory (ES) products and different functional proteins involved in the process of establishing infection have been investigated in rodent or swine models to explore their protective effect against *T. spiralis* infection. Moreover, different types of vaccines have been developed to improve the protective effect against *T. spiralis* infection in rodent or swine models, such as live attenuated vaccines, natural antigen vaccines, recombinant protein vaccines, DNA vaccines, and synthesized epitope vaccines. However, few studies of *T. spiralis* vaccines have been performed in pigs, and future research should focus on exploring the protective effect of different types of vaccines in swine models. Here, we present an overview of the strategies for the development of effective *T. spiralis* vaccines and summarize the factors of influencing the effectiveness of vaccines. We also discuss several propositions in improving the effectiveness of vaccines and may provide a route map for future *T. spiralis* vaccines development.

## Introduction

At present, the *Trichinella* genus comprises nine species and three genotypes that can be divided into encapsulated clade (*Trichinella spiralis*, T1; *Trichinella nativa*, T2; *Trichinella britovi*, T3; *Trichinella murrelli*, T5; *Trichinella nelsoni*, T7; *Trichinella patagoniensis*, T12; and *Trichinella* genotypes T6, T8, and T9) and non-encapsulated clade (*Trichinella pseudospiralis*, T4; *Trichinella papuae*, T10; and *Trichinella zimbabwensis*, T11) (Li et al., 2020; Feng et al., 2021). *T. spiralis* is an intracellular parasitic nematode that can infect a wide variety of mammals (Gottstein et al., 2009). Trichinellosis caused by *T. spiralis* is regarded as an emerging and re-emerging zoonotic parasitic disease in some parts of the world, such as in Argentina, Eastern Europe, and Asia (Cuperlovic et al., 2005; Devleesschauwer et al., 2017). Human can become infected with *T. spiralis* by ingestion of raw or poorly cooked meat containing the infective larvae of *T. spiralis* (Pozio, 2015). In humans, the clinical symptoms range from diarrhea and abdominal pain (intestinal phase) to fever, myalgia, myocarditis, and allergic reaction (muscular phase); in serious cases, facial edema and encephalitis may develop (Gottstein et al., 2009; Faber et al., 2015). Murrell and Pozio (2011) reported that 261 reports were selected from 494 reports for data extraction after applying strict criteria for relevance and reliability, and there are a total of 65,818 cases and 42 deaths were reported in 41 countries between 1986 and 2009. Although the husbandry condition of animals, meat inspection and public health and safety education have been enhanced to prevent trichinellosis, the International Commission on Trichinellosis (ICT) states that more than 11 million people are chronically infected with *T. spiralis* worldwide (Murrell and Pozio, 2011). China is one of the countries with the higher number of trichinellosis cases in the world. As the main source of human infection is domestic pigs and pork-related products, there is a need to develop an effective strategy to prevent *T. spiralis* transmission from pigs to humans (Cui et al., 2011; Cui et al., 2013a; Jiang et al., 2016). It is well-known that vaccines are among the most important ways to control diseases, and developing a vaccine against *T. spiralis* infection in pigs is a promising strategy to control *T. spiralis* infection.

To date, no effective vaccines are available to control *T. spiralis* infection, and therefore vaccine research should be intensified to prevent *T. spiralis* infection. The lack of progress in vaccine research against *T. spiralis* infection reflects both scientific obstacles, such as the complexity of life cycles and diversity of antigens, and policy deficiencies that have resulted in trichinellosis receiving little attention (Hewitson and Maizels, 2014). The developmental cycles of *T. spiralis* include three major stages: adult worms (AD), newborn larvae (NBL), and muscle larvae (ML) (Gottstein et al., 2009). Various antigenic components are expressed at each stage, and as such, a large number of antigens are available for vaccine research against *T. spiralis* infection. Furthermore, different life cycles of *T. spiralis* often occur in distinct tissues causing different immunological environments and pathological consequence. In general, vaccines should aim to block the development of infected larvae in the intestine, interrupt the growth of infected larvae to AD and expel AD from the intestine. Overall, the selection of adjuvants and the route of vaccination are associated with the effects of vaccines (Sander et al., 2020). Most studies of *T. spiralis* vaccines have been performed in murine models, whereas few studies have been conducted in swine models. Nevertheless, pigs are the natural host of *T. spiralis*, and studies have found that the immune responses induced by the same antigen are likely to differ between pigs and mice (Feng et al., 2013; Habiela et al., 2014; Xu et al., 2021). Feng et al. reported that mice vaccinated with a *T. spiralis* serine protease (rTs-Adsp) induced Th1-Th2 mixed immune response with Th2 predominant. However, Xu et al. reported that a Th1-Th2 mixed immune response with Th1 predominant was induced by rTs-Adsp after vaccination. Consequently, future studies of the development of *T. spiralis* vaccines should focus on pigs instead of mice.

Human trichinellosis is treated with anthelmintic drugs, such as mebendazole or albendazole. These drugs are considered relatively safe, except for pregnant women due to teratogenic effects (Shimoni and Froom, 2015). A vaccine for foodborne infection is the best method to control parasite infection, improve public health, and promote socioeconomic development (Hewitson and Maizels, 2014; McAllister, 2014). Compared with chemical treatment, vaccines have several advantages in the prevention of *T. spiralis* infection (Zhang N. et al., 2018). For example, vaccines provide long-term protection and eliminate the phenomenon of drug resistance of *T. spiralis*. Moreover, vaccines improve food safety by reducing drug residues in meat (Joachim, 2016; Sander et al., 2020). Studies have found that a window period between *T. spiralis* infection and anti-*Trichinella* IgG positivity (Gamble et al., 2004; Cui J. et al., 2015; Wang et al., 2017). There have been great efforts over the past few decades with respect to investigating the protective effect of *T. spiralis* vaccines through different strategies (Zhang N. et al., 2018). Vaccine development for *T. spiralis* infection is mainly divided into live attenuated vaccines, natural antigen vaccines, recombinant protein vaccines, DNA vaccines, and synthetic peptide vaccines. Although live attenuated vaccines elicit a strong immune response and protective immunity, their safety is doubtful. Inactivated vaccines are safe and have high protective effects, but antigen resources are limited due to the requirements of numerous animals. Currently, the development of *T. spiralis* vaccines focuses on recombinant vaccines and DNA vaccines. In this review, we will summarize the advances and future innovations in the development of *T. spiralis* vaccines as a strategy against trichinellosis.

## Vaccines Against *T. spiralis* Infection

**FIGURE 1:**
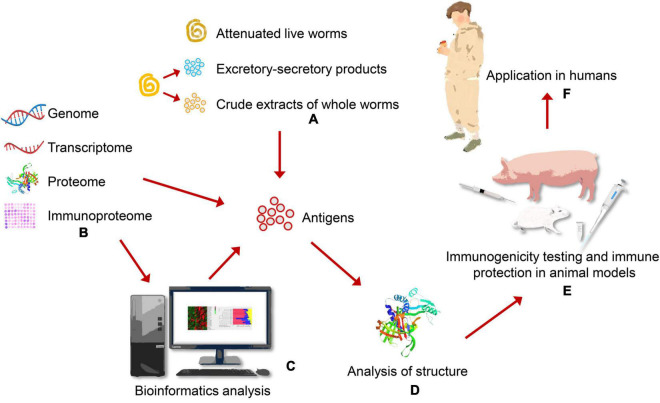
Discovery and identification vaccine candidates through different strategies in animal models. **(A)** Traditional *Trichinella spiralis* vaccines are derived from live attenuated vaccines, crude extracts of whole worms, excretory-secretory (ES) products. **(B)** Vaccine candidates can be screened through different strategies, including genome, transcriptome, proteome, immunoproteome. **(C)** Candidate antigens are selected for *T. spiralis* vaccines through bioinformatics analysis. **(D)** Analyzing structure of antigens contributes to designing effective vaccines in the future. **(E)** Immunogenicity and immune protection of vaccine candidates are identified through vitro and animal experiments. **(F)** High effective vaccines translate to meet people need and are applied in humans.

Traditional antigens of *T. spiralis* vaccines are derived from crude extracts of whole worms, excretory-secretory (ES) products. It is well-known that live attenuated vaccines and inactivated vaccines are first generation vaccines. With the development of genetic engineering methods, the strategy of genome, proteome, transcriptome, and immunoproteomics have been used to screen novel candidate antigens of *T. spiralis* vaccines (Figure 1). Based on these methods, second and third generation vaccines have been performed in rodent and swine models to evaluate their protective effect, such as recombinant protein vaccines, synthetic peptide vaccines and DNA vaccines. The strategies and protective effect of vaccines candidates against *T. spiralis* infection in different models are provided in Table 1.

**TABLE 1:** The protective effect of different type vaccines against *Trichinella spiralis* infection.

Vaccine type	Animal model	Antigen/Adjuvant	Antigen delivery	Dose	Protection	References
Live attenuated vaccines	Mice	Attenuated larvae	oral	300 attenuated larvae	72.5% reduction in ML	Hafez et al., 2020
Natural antigens vaccines	Pigs	Whole newborn larvae/Freund’s complete adjuvant	ip	3.5 × 10^5^ NBL	78% reduction in ML	Marti et al., 1987
	Mice	Larval Excretory-secretory (ES) products/Freund’s complete adjuvant	ip	10 μg	65.3% reduction in ML	Gamble, 1985b
	Mice	CTAB antigen/Freund’s complete adjuvant	sc	100 μg	50.42% reduction in ML	Grencis et al., 1986
Recombinant protein vaccines	Mice	*T. spiralis* serine protease (rTsSP)/cholera toxin subunit B	in	30 μg	71.10% reduction in Ad and 62.10% reduction in ML	Sun et al., 2019
	Mice	*T. spiralis* serine protease inhibitor (rTsSPI)/Freund’s complete adjuvant	sc	20 μg	62.2% reduction in Ad and 57.25% reduction in ML	Song et al., 2018
	Mice	*T. spiralis* adult-specific DNase II-1 (rTsDNase II-1)/Freund’s adjuvant	sc	20 μg	40.36% reduction in Ad and 50.43% reduction in ML	Qi et al., 2018
DNA vaccines	Mice	pcDNA3.1(+)-Ts-NBLsp	im	60 μg	77.93% reduction in ML	Xu et al., 2017a
Synthetic peptide vaccines	Mice	A 40-mer synthetic peptide	sc	100 μg	64.3% reduction in Ad	Robinson et al., 1995

*ip., intraperitoneal; in., intranasal; sc., subcutaneously; im., intramuscular. Not all antigens appearing in the article are listed in the table. Only representative antigens are listed in the table.*

### Live Attenuated Vaccines

Live attenuated vaccines were produced with radiation or drugs to reduce the pathogenicity of *T. spiralis* while maintaining immunogenicity. Experimental animals vaccinated with radiation-attenuated larvae exhibited an exciting reduction in muscle larvae burden (Cabrera and Gould, 1964; Agyei-Frempong and Catty, 1983; Hafez et al., 2020). For instance, Hafez et al. (2020) reported that mice vaccinated with radiation-attenuated larvae show a 72.5% reduction in muscle larvae burden, and Nakayama et al. (1998) found significant immunity in mice vaccinated with radiation-attenuated *T. britovi* larvae, with a strong worm reduction. Live attenuated vaccines perform high protective efficacy because they are similar to natural infection, mimicking an approximate microenvironment of *T. spiralis* infection. However, their safety is doubtful due to the potential of pathogenicity. The approach of live attenuated vaccines is gradually abandoned, even though it is associated with strong protective immunity.

### Natural Antigens Vaccines

#### Crude Extracts of Whole Worms

*Trichinella spiralis* has three major antigenic stages in the same host, namely, AD, NBL, and ML. Mice inoculated with adult worms soluble antigens showed an 89% reduction in adult worms and a 96% reduction in muscle larvae (Gamble, 1985a). Marti et al. (1987) reported that crude *T. spiralis* newborn larvae antigens were treated under freeze-thawing condition resulted in 78% muscle larvae reduction in pigs vaccinated with the antigens. Immunofluorescence results have shown that antibodies from the serum of immunized pigs bind to the surface of newborn larvae. Conversely, it was reported that soluble components of newborn larvae treated by ultrasonication have not protective effect against *T. spiralis* infection (Marti et al., 1987). Eissa et al. (2003) reported that mice vaccinated with the autoclaved *T. spiralis* larvae exhibit a significant reduction in adult worms and muscle larvae. The results of histopathological examination showed degeneration and hyalinization in the *T. spiralis* cyst wall accompanied by pericystic fibrosis. Another study found that the combination of antigens from adult worms and muscle larvae elicited a 73% reduction in *T. spiralis* female fecundity, a 96% reduction in adult worms and an 86% reduction in muscle larvae (Darwish et al., 1996). Although worm antigens from different stages induce a surprising protective effect, antigen resources are limited due to the requirement of numerous animals. Moreover, worm antigens are mainly presented *via* MHC-II pathway and mostly induce humoral immune responses (Aida et al., 2021). High effective vaccines usually induce strong humoral and cell-mediated responses to expel parasites from the host’s intestine. With the development of technology, second and third generation vaccines spring up due to these disadvantages of inactivated vaccines.

#### Excretory-Secretory Products

The major origin of ES antigens are the stichosome and stichocytes (Gamble et al., 1983; Gamble et al., 1988). Here, we summarize the protective effect induced by crude ES products and components of ES products with a defined molecular size. After *T. spiralis* infection, ES antigens are directly exposed to the immune system of the host, and they are the target antigens that induce an immune response of host (Lightowlers and Rickard, 1988). Thus, ES antigens play a critical role in diagnosing and preventing trichinellosis (Todorova, 2000; Korinkova et al., 2008). According to Isao et al., the main components of ES products in muscle larvae consist of 43 kDa, 53 kDa, and 45 kDa glycoproteins, which are derived from stichosomes (Nagano et al., 2009). Studies on ES products of *T. spiralis* contribute to understanding the interaction between host and parasite, and in developing vaccines against *T. spiralis* infection.

Gamble et al. (1986) reported that pigs vaccinated with the ES antigen of muscle larvae had a significant reduction in female fecundity and a 57% reduction in muscle larvae. Moreover, Gamble (1985b) found significant levels of protection in mice immunized with an antigen isolated from ES products. Although ES antigens keep natural activity and have excellent immunogenicity, ES antigen resources require large numbers of animals. With the development of science and technology, some new strategies have been applied to screen antigens in ES products, such as proteomics and immunoblotting (Liu et al., 2016a,b), and in most studies, the antigen genes screened from ES products are cloned and expressed in a prokaryotic expression system. In the future, more potential immunogenic antigens should be selected from among ES products, and the protective effect of these antigens should be further explored.

#### Surface Antigens

*Trichinella spiralis* surface proteins are directly exposed to the immune system of the host and may play an important role in the process of *T. spiralis* invasion (Cui et al., 2013b; Liu R. D. et al., 2014). Grencis et al. (1986) reported that several surface antigens are stripped from the cuticle of *T. spiralis* larvae and that the relative molecular masses of the antigens are 100 kDa, 90 kDa, 69 kDa, 55 kDa, 46 kDa, and 35 kDa. Moreover, mice immunized with surface antigens showed a reduction in adult worms, muscle larvae and female fecundity. In the study of Ortega-Pierres et al. (1989), several antigens extracted from *T. spiralis* larvae displayed the same structural features and surface antigens conferred partial protection against *T. spiralis* infection in mice. Aquaporins (AQPs), also known as water channel proteins, are components of many parasite membranes (Benga, 2009; Faghiri and Skelly, 2009). An aquaporin gene from *T. spiralis* (TsAQP) has been identified, and the TsAQP protein is predicted to contain six linear B-cell epitopes, suggesting it is a promising antigen for vaccines (Cui J. M.et al., 2015).

Surface antigens of *T. spiralis* are complex, because they may change during the process of worm molting and growth (Bolas-Fernandez and Corral Bezara, 2006). Surface antigens comprise a series of proteins of different biological processes, such as immune reactions, adhesion molecules, and enzyme (Cui et al., 2013b). These antigens are important modulators of the host immune system, and studies of intestinal immunity have revealed that these antigens play a critical role during the process of *T. spiralis* invasion and development. Surface antigens of *T. spiralis* have been identified and characterized by two-dimensional gel electrophoresis (2-DE), mass spectrometry and immunoproteomics (Cui et al., 2013b; Liu R. D. et al., 2014). Research on surface antigens contributes to understanding the host-parasite interaction and identifying target antigens for detection, diagnosis and vaccine development (Bolas-Fernandez and Corral Bezara, 2006; Cui et al., 2013b; Liu R. D. et al., 2014).

### Recombinant Protein Vaccines

Certain progress in the development of recombinant protein vaccines against *T. spiralis* infection has been achieved with the rapid development of genetic engineering. Candidate antigens have mainly been screened from ES products, functional proteins and antigens involved in the processes of *T. spiralis* invasion. Protease and protease inhibitor are the most important components of ES products involved in *T. spiralis* infection. In recent years, a large amount of protein vaccine research has been performed on serine proteases, serine proteases inhibitors, cystatins and deoxyribonuclease to control *T. spiralis* infection.

#### Proteases

Serine proteases from ES products are thought to be key factors in *T. spiralis* invasion of host cells and in processes of immune evasion (Dzik, 2006). Sun et al. (2019) reported that mice vaccinated with recombinant *T. spiralis* serine protease (rTsSP) showed 71.10 and 62.10% reduced worm burdens of AD and ML, and Wang et al. (2013) reported that mice immunized with recombinant *T. spiralis* serine protease (rTspSP-1.2) displayed 34.92 and 52.24% reduced worm burdens of AD and ML. A previous study in our laboratory has found that mice vaccinated with recombinant *T. spiralis* serine protease (rTs-Adsp) exhibited a 46.5% reduction in muscle larvae (Feng et al., 2013). Further research showed a 50.9% reduced worm burden of ML in pigs immunized with rTs-Adsp (Xu et al., 2021). Furthermore, cysteine proteases of parasites are also a focus of attention for parasite vaccines. Cysteine proteases are essential hydrolases present in most organisms, such as viruses and parasites. Cysteine proteases of parasites play an important role in invasion of host tissue and maintenance of parasite survival in the host, rendering them a major target for the development of parasite vaccines (Grote et al., 2018).

#### Proteases Inhibitor

Serine proteases inhibitors (serpins) are a superfamily of proteins that suppress the activity of serine proteases and play an important role in blood coagulation, inflammation, and complement activation (Molehin et al., 2012). The serpins secreted by worms protect them from the serine proteolysis of the host, and thus, they help parasites overcome defensive barriers and avoid host immune attack (Dzik, 2006). Mice vaccinated with recombinant *T. spiralis* serine protease inhibitor (rTsSPI) exhibited 62.2 and 57.25% reduced worm burdens of AD and ML, in the study by Song et al. (2018), and a 59.95% reduction in adult worms at 10 days post-infection (dpi) and a 46.41% reduction in muscle larvae were detected in mice immunized with recombinant *T. spiralis* serpin (rTs-Serpin) according to Xu et al. (2017b). Cystatins are a superfamily of proteins that specifically inhibit cysteine protease activity (Brown and Dziegielewska, 1997). In nematodes, cystatins play a crucial role in immune evasion and regulate the host immune response during parasite infection (Vray et al., 2002; Hartmann and Lucius, 2003). Tang et al. (2015) reported that mice vaccinated with a cystatin-like protein (Ts-CLP) exhibited a 64.28% reduction in adult worms at 5 dpi and a 61.21% reduction in muscle larvae. Liu X. D. et al. (2014) found that oral administration of a cystatin-like protein to mice resulted in a 91% reduction in the parasite female fecundity.

#### Deoxyribonuclease II

DNase II mainly exists in lysosomes and nuclei, and plays an important role in pathogen invasion and evasion of the immune response of the host. Compared to DNase II in other species, the DNase II protein family of *T. spiralis* has significantly enlarged. Moreover, studies have found that DNase enzymes of *T. spiralis* may play a key role in parasite-host interactions during infection, suggesting that they can be used as candidate antigens to control and prevent trichinellosis (Liu R. D. et al., 2015; Liu et al., 2016b). Qi et al. (2018) reported that mice subcutaneously vaccinated with rTsDNase II-1 and rTs-DNase II-7 showed 40.36 and 34.86% reductions in adult worms at 5 dpi and 50.43 and 42.33% reductions in muscle larvae, respectively. A previous study by our laboratory found that pigs vaccinated with DNase II-7 recombinant protein showed a 45.7% reduction in muscle larvae (Xu et al., 2020b). Although recombinant protein vaccines have become increasingly popular, the level of immunoprotection is still associated with the antigens, adjuvants, and delivery routes.

### DNA Vaccines

DNA vaccines have become more attractive due to their ability to induce a broad immune response and long-lasting immunity (Gu et al., 2014). Additionally, compared to conventional protein vaccines, DNA vaccines are much more stable, cost efficient, simple to produce, and safe for use (Prazeres and Monteiro, 2014; Kumar and Samant, 2016). Compared to protein vaccines, the main disadvantage of DNA vaccines is poor immunogenicity (Li et al., 2012). Furthermore, designing a vaccine suitable for humans is a great challenge for DNA vaccines against parasites. Many studies of DNA vaccines against parasites are focused on murine models, but the results cannot be applied in humans (Kumar and Samant, 2016).

Many DNA vaccines against *T. spiralis* infection have recently been developed in murine models (Gu et al., 2014; Xu et al., 2017a,2020a). In the study of Wang L. et al. (2016), mice vaccinated with a *Salmonella*-delivered *TsPmy* DNA vaccine showed 44.8 and 46.6% reduced worm burdens of AD and ML, respectively; Xu et al. (2017a) found a 77.93% reduced worm burden of ML in mice vaccinated with the pcDNA3.1(+)-Ts-NBLsp DNA vaccine. Overall, compared to protein vaccines, the immunogenicity of DNA vaccines is poor due to low levels of antigens expression. Studies have found that DNA plus protein vaccination is an ideal strategy for improving the immune response and protective effect. Gu et al. (2014) reported that mice immunized with Ts87 in a DNA-prime/protein-boost strategy experienced a 46.1% reduced worm burden of ML. In a previous study in our laboratory, mice immunized with pcDNA3.1(+)-Adsp/rTs-Adsp exhibited a 69.50% reduced worm burden of ML (Xu et al., 2020a). With the rapid development of technologies, more methods will be performed to enhance the efficacy of DNA vaccines. The ultimate goal is to develop a DNA vaccine that can be safely applied in humans.

### Synthetic Peptide Vaccines

In the past several decades, much research has been carried out to develop vaccines against *T. spiralis* infection, such as crude antigens, recombinant proteins, and DNA vaccines. However, there are only a few studies on controlling *T. spiralis* infection utilizing peptide vaccines. Compared to recombinant protein vaccines, multiepitope peptides have advantages of being easy and fast to produce and may include of multiple protective epitopes (Gu et al., 2020). Robinson et al. (1995) screened a 40-mer synthetic peptide from the glycoprotein of *T. spiralis*, and mice vaccinated with the peptide vaccine exhibited a 64.3% reduction in adult worms, and McGuire et al. (2002) reported a 33.3% reduction in parasite female fecundity in mice immunized with a 30-mer peptide antigen.

In recent years, epitope vaccines against viral, bacterial or even parasitic infection have been rapidly developed. However, epitope vaccines also have some disadvantages: they have poor immunogenicity and need to be conjugated to a large carrier protein. A new strategy of multiple antigenic peptides was developed to improve the immunogenicity of epitope vaccines. Epitope-based vaccines can be constructed as chimeric vaccines by engineering multiple effective epitopes (Gu et al., 2013). Consequently, a chimeric vaccine may improve the level of epitope vaccine protection or prevent parasite immune evasion. Moreover, the life cycle of *T. spiralis* is complex leading to various antigens at different stages. A multiepitope vaccine is an effective way to control *T. spiralis* infection.

## Factors of Vaccine Effectiveness

**FIGURE 2:**
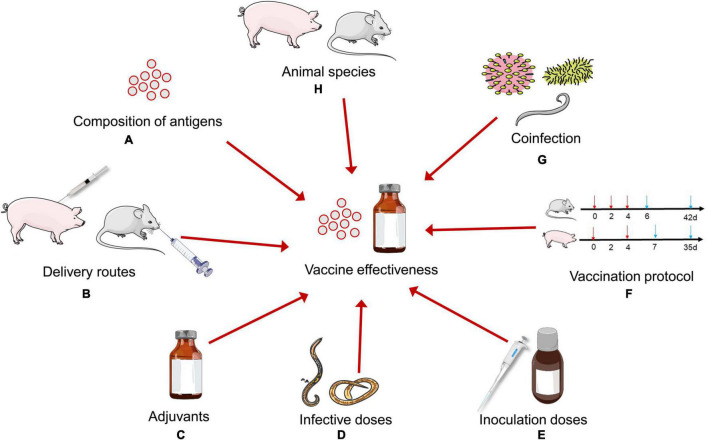
Factors influencing the effectiveness of *Trichinella spiralis* vaccines in animal models. **(A)** Various candidate antigens show different protective effects. **(B)** Several vaccine delivery routes have been used to improve the effectiveness of *T. spiralis* vaccines. **(C)** Suitable adjuvants play a very important role in vaccines effectiveness. **(D)** Infective doses of *T. spiralis* may determine the effectiveness of *T. spiralis* vaccines in animal models. **(E)** The efficiency of immunization also depends on inoculation doses of vaccines. **(F)** There is a lack of a standardized vaccination protocol in animal models. **(G)** Coinfection change the host’s immune response to affect the effectiveness of *T. spiralis* vaccines. **(H)** The same antigen may exhibit different protective effects in different animal models.

There are many factors that influence the effectiveness of vaccines, such as the composition of antigens, adjuvants, inoculation doses, delivery routes, infective doses, coinfection, animal species, and vaccination protocol (Figure 2). *T. spiralis* has a multistage life cycle, resulting in different antigens at different stages. The composition of antigens determines the effectiveness of vaccines thus identification of excellent antigens is crucial for developing *T. spiralis* vaccines. Different candidate antigens elicit distinct immune response and protective effects. During *Trichinella* infection, Th2-type cytokines are secreted by hosts to promote mast-cell activation and proliferation, which are important for expelling parasite from intestine (Zhang N. et al., 2018). Future studies of the development of *T. spiralis* vaccines should focus on the antigens that could elicit Th2-type immune response. Selecting a suitable adjuvant plays a very important role in vaccine development (Burakova et al., 2018). Adjuvants enhance immune responses induced by parasite antigens, and protect the antigens from being diluted, degraded and eliminated by the host (Stills, 2005). To date, the adjuvants used in experiments include Freund’s adjuvant, aluminum hydroxide and emulsions containing water in oil, oil in water, and multiphasic formulations. Freund’s adjuvant has been thought to be the gold standard adjuvant and has been used widely in many studies. Nevertheless, the application of Freund’s adjuvant is gradually limited due to its toxic effect and specific damage to experimental animals. Although few adjuvants surpass Freund’s adjuvant in inducing antibody production, many adjuvants can also induce high antibody responses with less inflammation and tissue destruction. In recent decades, alternative adjuvants have been evaluated against *T. spiralis* infection in murine models, including Montanide ISA series adjuvants and Montanide™ IMS series adjuvants. Montanide™ IMS series adjuvants containing water-dispersed liquid nanoparticles combined with an immunostimulating compound are comparatively non-toxic and have been employed in research on *T. spiralis* vaccines (Jang et al., 2011; Xu et al., 2017b). Yang et al. (2010) reported that recombinant Ts-Pmy formulated with Montanide ISA206 or ISA720 induced similar levels of immune responses and protection, compared to that of Freund’s adjuvant formulation group.

Although the adjuvant is very important, the efficiency of immunization also depends on inoculation doses. Regarding dose, Gamble (1985a) reported that mice immunized with 10 μg antigen exhibited a 77% reduction in muscle larvae but that mice immunized with 100 μg antigen displayed a 98% reduction in muscle larvae. In most research, the dose of vaccination in a murine model has varied from 20 to 100 μg. Moreover, few studies have been performed in pig to explore the dose of vaccination and hence there are no standard inoculation doses in pig models. Although mice were immunized with the same antigen formulated with different adjuvants and delivery routes, the immune protection induced differed. At present, vaccine delivery routes include subcutaneous, intradermal, intraperitoneal, intramuscular, intranasal, and oral inoculation. The strategy of encoding *T. spiralis* antigens by the attenuated *Salmonella* and recombinant *Lactobacillus plantarum* has been widely used in the development of *T. spiralis* vaccines, which have been proven to induce mucosal immunity (Liu X. D. et al., 2014; Liu P. et al., 2015; Wang L. et al., 2016; Hu et al., 2021). The model of *T. spiralis* infection involves the intestinal mucosa, and its target is to induce a local immune response. Studies have found that oral or intranasal vaccination can activate systemic and mucosal immune responses. Therefore, vaccination through oral or intranasal routes can be effective, inducing a broad mucosal immune response (Ortega-Pierres et al., 2015).

In most research, the dose of infection in a murine model has varied from 200 to 400 *T. spiralis* ML. In a pig model, Marti et al. (1987) reported that pigs were challenged 2000 *T. spiralis* ML to evaluate the protective effect of antigen. However, Xu et al. (2020b,2021) reported that pigs were inoculated with 5000 *T. spiralis* ML by oral administration to evaluate the effectiveness of vaccines. The effectiveness of the same candidate antigen may be influenced by infective dose in animal models. Moreover, whether or not the dose of infection is similar to natural infection remains to be explored. In the natural setting, bacteria, viruses and parasites may exist in one host (Wang et al., 2019). Coinfection may change the host’s immune response to affect the effectiveness of *T. spiralis* vaccines. Whether or not immune response induced by *T. spiralis* vaccines can be suppressed or neutralized by other pathogens infection remains to be determined. Understanding the immune response induced by multiple pathogens should be a part of *T. spiralis* vaccines development in future. It is well known that animal models play crucial roles in developing vaccines. Most studies on protective effect of vaccines have focused on murine models rather than on pig models. However, the effectiveness of vaccines may vary depending on animal species. Previous studies of our laboratory have found that immune response induced by the same antigen is different between pigs and mice (Feng et al., 2013; Xu et al., 2021). Therefore, high levels of protection induced by candidate antigens in murine models should be further verified in swine models. Considerable efforts have been made to design and verify various vaccination strategies. Although there have been mature vaccination regimens in murine models, they may not be available for pigs or humans. So far, there has been a lack of a standardized protocol in swine models. Consequently, the factors influenced the effectiveness of vaccines should be strongly considered for *T. spiralis* vaccines development in future.

## Future Perspectives and Challenges

Although substantial efforts and progresses have been made to search for candidate antigens and develop *T. spiralis* vaccines, there is still no effective vaccines to prevent *T. spiralis* infection. With the development of genomics, proteomics, transcriptomics, more immunogenic antigens have been isolated and identified to develop effective *Trichinella* vaccines. More and more strategies have been applied to increase the effectiveness of vaccines. Currently, DNA vaccines are becoming more appealing due to their several advantages, such as cost-effectiveness, stable, long-lasting immunity. Regarding toxoplasmosis vaccines, a DNA multicomponent vaccine showed an 80.22% reduction in the parasite cyst burden (Zhang N. G. et al., 2018). The cocktail DNA vaccine may be a promising strategy to improve the effectiveness of *T. spiralis* vaccines. Moreover, combination vaccination has been used as a promising approach to improve the effectiveness of *T. spiralis* vaccines in murine models. The strategy of DNA plus protein vaccination exerts the advantages of DNA and protein vaccines to induce high levels of immune response and immune protection. Virus-like particle (VLP) vaccines are one of most popular strategy due to their ability to induce strong immune responses (Pitoiset et al., 2015). The approach has been applied in the field of toxoplasmosis vaccines, and provides a novel idea for the development of *T. spiralis* vaccines. With the development of genetic engineering approaches, the strategy of gene editing has been used to develop live-attenuated toxoplasmosis vaccines (Wang J. L. et al., 2016). Although physiological characters are different between *T. spiralis* and *Toxoplasma gondii*, the strategy can be used as a reference for developing an attenuated *Trichinella* vaccine.

The life cycle of *T. spiralis* in the host is complex, including a diversity of antigens, immune evasion, and regulation of host response. These characteristics make it difficult to achieve the ideal protection with a single *T. spiralis* antigen. The ideal vaccine should promote the process of expelling AD and inhibit the production of NBL and the formation of ML. Moreover, the immune response induced by vaccines should have the ability to disable, degrade, and dislodge the parasites (Maizels et al., 2012). With the development of genetics, new research ideas are being proposed to improve the protection rate of vaccines against *T. spiralis* infection. In-depth research on immunosuppression and immune evasion caused by *T. spiralis* infection will contribute to the design of more effective vaccines against *T. spiralis* infection.

The main source of human *T. spiralis* infection is pork and pork-related products. To date, most research on *T. spiralis* vaccines has been performed in murine models, and more research in pigs should be conducted. The financial and technical issues associated with *T. spiralis* vaccines using swine models make research difficult, but this is a key factor in many vaccinology studies. As the risk of livestock infection with *T. spiralis* is negligible under reasonable management condition, the development of vaccines against *T. spiralis* infection has received little attention. Regardless, vaccines against *T. spiralis* infection are a safe tool that could avoid drug resistance. Therefore, it is crucial to educate the public on the importance of vaccination and its benefit.

## Conclusion

A successful vaccine for trichinellosis depends on a thorough consideration of immune response caused by *T. spiralis* infection and the factors that influence the effectiveness of vaccines. Finally, research on the protective effect of *T. spiralis* vaccines should focus on pig infection models. To conclude, vaccines are a promising strategy to control trichinellosis.

## Author Contributions

DX, BT, and TL provided the ideas and wrote the draft manuscript. JL, YX, WG, YZ, and SY contributed to the revising of the manuscript. ML and DX approved the version to be published. All authors read and approved the final manuscript.

## Conflict of Interest

The authors declare that the research was conducted in the absence of any commercial or financial relationships that could be construed as a potential conflict of interest.

## Publisher’s Note

All claims expressed in this article are solely those of the authors and do not necessarily represent those of their affiliated organizations, or those of the publisher, the editors and the reviewers. Any product that may be evaluated in this article, or claim that may be made by its manufacturer, is not guaranteed or endorsed by the publisher.
